# Engaging Men in Prevention and Care for HIV/AIDS in Africa

**DOI:** 10.1371/journal.pmed.1001167

**Published:** 2012-02-07

**Authors:** Edward J. Mills, Chris Beyrer, Josephine Birungi, Mark R. Dybul

**Affiliations:** 1 Faculty of Health Sciences, University of Ottawa, Ottawa, Canada; 2 Center for Public Health and Human Rights, Department of Epidemiology, Johns Bloomberg School of Public Health, Baltimore, Maryland, United States of America; 3 The AIDS Support Organization (TASO), Kampala, Uganda; 4 O'Neill Institute for National and Global Health Law, Georgetown University, Washington, D.C., United States of America; 5 George W. Bush Institute, Dallas, Texas, United States of America

## Abstract

Ed Mills and colleagues argue that a more balanced approach to gender is needed so that both men and women are involved in HIV treatment and prevention.

**Summary Points**

- The HIV/AIDS response in Africa has always had a gender focus; targeted efforts have reduced the impact of the epidemic on women and children.
- The response has been far less successful for the treatment of men: there is less ART coverage of men than women in Africa, and men typically have higher mortality. Men also tend to present at clinic with advanced disease and are more likely to be lost to follow-up.
- Yet, efforts to understand men's healthseeking behaviour are poorly understood in the AIDS epidemic, and encouraging men to get tested and treated is a major challenge, but one that is poorly recognized.
- We review the emerging evidence and we call for a balanced approach to gender programming in an effort to involve both men and women in treatment and prevention.

Antiretroviral therapy (ART) saves lives and prevents new HIV/AIDS infections [1]. Successful efforts to increase the number of people receiving ART create important public health challenges, some of which may be considered counter-intuitive. One of the largest challenges for ART provision has been targeting populations most affected by HIV/AIDS and most vulnerable to the effects of the disease. In Africa, the focus of the epidemic has historically been on women and children. Women are considered to be particularly vulnerable to HIV infection in this setting because of biological factors, their reduced sexual autonomy, and men's sexual power and privilege over them. [2]–[6]. This understanding has led HIV/AIDS public health prevention and treatment campaigns to focus on women and children in this setting. As a result, men have received considerably less attention in the epidemic [7] and receive less targeted HIV prevention and treatment programs [5].

Targeting men in prevention and treatment, however, may have a large impact on mortality, new infections, and the economic impact of HIV/AIDS in Africa. In the wake of the HPTN 052 trial results, demonstrating 96% (95% confidence interval, 73%–99%) efficacy of prevention in discordant couples with earlier ART treatment initiation, engaging greater numbers of men with HIV in treatment could have important prevention benefits for women and girls, and for primary prevention of vertical transmission [1].

## Neglect of Men in HIV Prevention and Treatment Campaigns

In the last half-decade, there has been discussion over the need to actively engage men in sub-Saharan Africa in HIV prevention campaigns. Several randomized trials in South Africa have examined interventions aimed at male behavior change [8]–[12]. Further work has come from the social science disciplines, where researchers and gender advocates have created gender-focused HIV prevention frameworks and contextualized the role of men in contributing to the epidemic [5],[13],[14]. Although much of this work has examined attitudes and behaviors, there is emerging recognition from a number of epidemiological sources that men in sub-Saharan Africa face important challenges in terms of HIV vulnerability, engagement and retention in care, and access to ART that affect mortality [15],[16]. Taken together, the evidence indicates that men are under-represented in HIV testing, treatment, and care, and this likely has a direct impact on outcomes of care [17]–[21].

While public health efforts have been aimed at women, particularly child-bearing women (e.g., HIV testing, care, and treatment opportunities provided through antenatal care services), scale-up efforts are hindered by the differences in health-seeking behaviors between men and women [22]. For instance, sickness may be seen as a sign of weakness for many men, and this perception has resulted in a reluctance of care-seeking among men [23]. There is also evidence indicating that men may feel that they have been caught at their hidden sexual behaviors and so they avoid HIV testing [23]. Additionally, employment-related migration will keep men away from their partner and families for long time periods, and this absence may make them more vulnerable to HIV infection due to sexual exposure, drug and alcohol use, and delinkages with local health services [22]. The reality that men are less likely to seek health care is intimately linked to perceptions of masculinity, and is generally considered to be part of the same phenomenon that drives multiple partnering, violence against women, substance use, and homophobia among men [5],[13].

There is now also a growing appreciation that the HIV/AIDS epidemic in Africa is driven by complex and poorly understood sexual dynamics that include, among others, concurrent partner relationships and multiple partner relationships involving both males and females [24]–[26]. The available evidence indicates that infection is equally balanced between males and females in most heterosexual settings [25].

Failing to engage men in HIV prevention and treatment may also have an impact on household family income. In Africa, men are typically the larger income-generators, often engaged in employment outside of the home, whereas women are more likely to be engaged in economic activities closer to home as well as child caring. If the head male member contracts HIV and does not receive the appropriate care, ill health or death of this individual can severely impact household family income.

While our discussion here is predominantly focused on heterosexual men, we cannot ignore that men who have sex with other men (MSM) are one of the most difficult groups to target in prevention and treatment campaigns in Africa. Data on the magnitude of MSM or the prevalence of HIV in this population are sparse [27]. The recent crackdown on MSM in Uganda, where the government petitioned a law before parliament to make MSM sexual activities illegal, potentially punishable by death for those who are HIV positive, demonstrates that certain male groups require specific care and support [28]. The law, largely condemned around the world, also placed pressure on HIV/AIDS service providers, as anyone, including organizations, aware of homosexual activity and failing to report the act could be punished with up to three years of imprisonment. With the popular support the bill has received, HIV/AIDS service organizations have been challenged to provide strong advice to their employees on how to treat MSM patients. Similar legal and cultural oppression of MSM occurs in other African countries.

## The Magnitude of HIV/AIDS-Related Mortality by Gender

The gender differences inherent in the health-seeking behaviors of men and women, and the historical gender-specific efforts in HIV-related public health campaigns in this region, impact health outcomes, including mortality [16],[17],[29]. For instance, recent cohort studies conducted among individuals starting ART in sub-Saharan Africa have indicated that men tend to access ART at a later disease stage than women, and the risk of mortality once on ART is much higher for men than women, even when adjusting for disease state [15],[30]. Specifically, in Uganda, evidence from a large, nationally representative cohort study indicates that men are (hazard ratio, HR) 1.43 (95% confidence interval: 1.31–1.57) times more likely to die than women [30], and in South Africa, evidence from a large cohort study indicates that men are 1.47 (HR, 95% confidence interval: 1.27–1.72) times more likely to die than women [15]. Using these estimates, and demographic input assumptions and population estimates [31],[32], HIV prevalence [33]–[35], and the number of individuals receiving ART [36]–[40], we can develop a simple projection model to estimate HIV/AIDS-related mortality by gender for the two counties. Assuming that these estimates remain constant, a crude mortality projection from 2004–2015 indicates that the cumulative number of national HIV/AIDS-related deaths for those aged 15–49 years is much higher among males when compared to females in both Uganda (475,986 cumulative number of deaths for males versus 204,674 cumulative number of deaths for females) and South Africa (2,488,286 cumulative number of deaths for males versus 1,169,494 cumulative number of deaths for females). (Please contact the primary author for a complete description of the model assumptions.)

## Targeted Prevention

Although there have been efforts to involve men at antenatal clinics, these have had mixed results in terms of HIV prevention [41]. There are examples of HIV prevention programs in Africa that have intentionally targeted men in their campaigns to change sexual behaviors [42]–[46]. However, they are predominantly concerned with primary prevention, and rarely consider treatment interventions. A small body of evidence is emerging indicating that programs integrated into the workplace and programs that offer peer education may be successful at engaging African men in HIV testing, care, and treatment [47].

Funding agencies should recognize that males and females are both severely affected by the epidemic in differing ways, and should plan for interventions that engage both men and women. Funding agencies, such as the US President's Emergency Plan for AIDS Relief (PEPFAR), frequently allocate funding according to priority groups, particularly women and children [48]. Targeted and sustainable funding may result in important lessons learned.

Targeting specific populations for ART treatment and care can have important residual effects on preventing transmission to other populations. The HPTN 052 trial confirmed findings from observational studies that ART has a large preventive impact on sexual partners [1]. Given the economic reality, scaling up access to ART as a prevention strategy will be a challenge due to costs, human resources constraints, and prioritizing recipients; targeting those individuals and groups who are most likely to transmit the virus, core transmitters, may be a first step in using ART treatment as prevention in a scaled up manner. It is also likely that male circumcision clinics, slowly growing in number in sub-Saharan Africa, would be ideal venues to test men for HIV, and provide them with appropriate referrals for care and treatment. Mobile approaches to testing targeted at venues, including work spaces, frequented by men may also have significant impacts on increasing male engagement in prevention and treatment. This approach has been demonstrated by the success of HPTN 043 (Project Accept) reported in 2011, that markedly increased male acceptance of testing in four countries (South Africa, Zimbabwe, Tanzania, and Thailand) [49]. Targeted treatment of all individuals who are HIV positive and in a relationship or sexually active will reduce their viremia and reduce their potential to infect others [50].

The epidemiological evidence is accumulating, and indicates that males in sub-Saharan Africa are not accessing HIV services as often as their female counterparts, and as a result, men have worse outcomes of care, including mortality. Funding organizations need to recognize the social and health impacts associated with not engaging men in primary and secondary HIV prevention campaigns. Programmatic efforts should account for this disparity, and recognize that it may be necessary to seek out men for HIV testing, care, and ART in variety of settings, and through mechanisms that that take into consideration the local culture and gender roles in partnerships, sex, and health.

## Abbreviations

ART: antiretroviral therapy
MSM: men who have sex with other men
